# Paraneoplastic hypoglycemia in a patient with a malignant solitary fibrous tumor

**DOI:** 10.1530/EDM-14-0026

**Published:** 2014-05-01

**Authors:** Kamel Mohammedi, Charbel Abi Khalil, Sophie Olivier, Imane Benabad, Ronan Roussel, Michel Marre

**Affiliations:** 1 Assistance Publique Hôpitaux de ParisBichat Hospital, Department of Diabetology, Endocrinology and Nutrition46 rue Henri Huchard75877, Paris Cedex 18France

## Abstract

Hypoglycemia is a common medical emergency. It is the most frequent complication induced by anti-diabetic treatment. However, it can be observed in other conditions unrelated to diabetes such as insulinoma, autoimmune disorders, and neoplasia. Herein, we report the case of a rare cause of severe and recurrent hypoglycemia in a 77-year-old woman with a malignant solitary fibrous tumor (MSFT). A 77-year-old woman was admitted to the emergency department for loss of consciousness induced by severe hypoglycemia. Her standard laboratory findings were unremarkable. HbA1c, albumin, renal, liver, thyroid, and adrenal function tests were normal. Cerebral CT scan was also normal. At the time of confirmed hypoglycemia, the serum level of insulin and C-peptide was low. On the basis of the past medical history and the absence of other comment etiologies, a paraneoplastic cause was suspected. Thus, the diagnosis of a non-islet cell tumor-induced hypoglycemia (NICTH) was established by the presence of incompletely processed precursors of IGF2 (big IGF2) in plasma electrophoresis. However, the IGF1 level was low. Therapy with corticosteroids improved hypoglycemia and clinical symptoms. NICTH is a rare cause of hypoglycemia. It should be considered in patients with mesenchymal or malignant epithelial tumors suffering from recurrent episodes of hypoglycemia. The diagnosis will be established in the case of low serum insulin concentrations and elevated levels of big IGF2. Treatment with corticosteroids, GH, or both can improve hypoglycemic symptoms and restore plasma glucose to normal levels.

**Learning points:**

- NICTH is a very rare condition that should be considered in patients known to have mesenchymal or malignant epithelial tumors and suffering from recurrent episodes of hypoglycemia.
- The diagnosis of an NICTH is established on the basis of the hypoinsulinemic hypoglycemia, the MSFT history, and the presence of paraneoplastic secretion of IGF1 or an immature form of IGF2.
- Treatment with corticosteroids, GH, or both can improve hypoglycemic symptoms and restore plasma glucose to normal levels in NICTH.

## Background

Hypoglycemia is a common medical emergency in diabetic patients treated with insulin or oral hypoglycemic drugs. However, it can be observed less frequently in other conditions such as insulinomas and rare autoimmune diseases (1). Paraneoplastic disorders are an exceptional etiology of hypoglycemia. In this case, paraneoplastic secretion of insulin-like growth factor 1 (IGF1) or partially processed precursors of IGF2 could be responsible for hypoglycemia (2) (3). Herein, we report the case of severe and recurrent hypoglycemia in a woman with a malignant solitary fibrous tumor (MSFT).

## Case presentation

A 77-year-old woman was admitted to the emergency department for loss of consciousness. Laboratory measurements showed very low plasma glucose levels (21 mg/dl). The patient was not diabetic and she did not take any glucose-lowering drugs. Her previous treatment comported with propranolol 40 mg, levothyroxine 100 μg, zopiclone 3.75 mg, lactulose 10 g, calcium 1000 mg, and colecalciferol 880 UI. She was treated according to standard hypoglycemia protocols and transferred to the endocrinology department.

In her past medical history, we noticed the presence of hypertension, hypothyroidism following thyroidectomy, and an MSFT, for which she had undergone to surgery and chemotherapy. Nevertheless, none of the curative treatment of the MSFT could be considered.

The patient reported a previous feeling of faintness with symptoms of hypoglycemia few days before her admission to the emergency department. During hospitalization, hypoglycemic episodes occurred at any time, with a much higher severity on waking and before meals. Her symptomatology was particularly characterized by neuroglycopenic signs with fatigue, weakness, headache, dysphasia, and loss of consciousness, without seizures. The Glasgow coma scale was 9.

## Investigation

**Figure 1:**
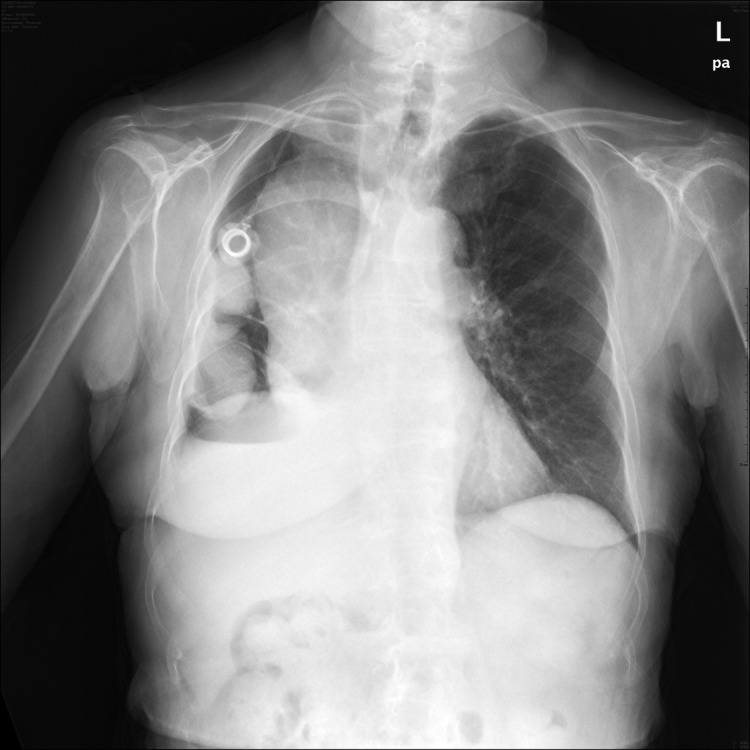
Chest X-ray of a 77-year-old female with a malignant solitary fibrous tumor showing three large masses in the right pleural muscles.

During hospitalization within the Department of Endocrinology, her weight was 50 kg, her height 155 cm, her blood pressure 105/68 mmHg, and her pulse 74/min. HbA1c was 5.5%. Albumin, renal, and liver function tests were normal. Plasma cortisol levels were 340 and 1680 nmol/l before and 60 min after injection of 250 μg tetracosactrin respectively. TSH was 4.34 mUI/l (normal range: 0.25–4.5) and thyroid hormones were normal. The diagnosis of insulinoma was unlikely according to the serum level of insulin and C-peptide <1 μU/ml (normal <13) and 0.18 ng/ml (normal <3.2) respectively. Cerebral CT scan was normal. The chest X-ray showed three large masses in the right pleural muscles (Fig. 1).

**Figure 2:**
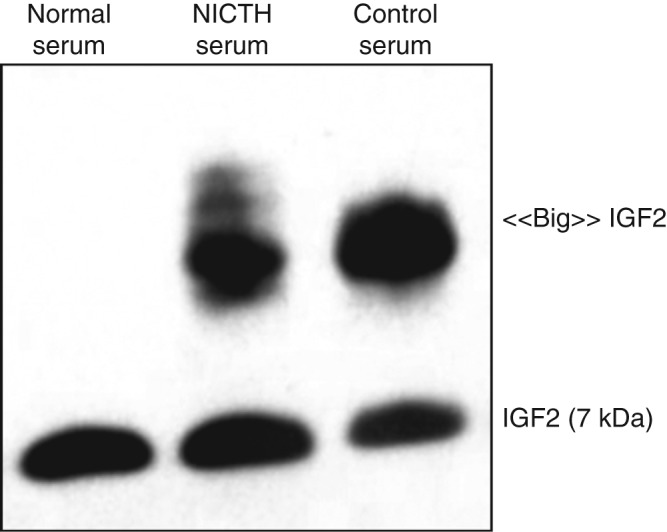
Electrophoresis of plasma IGF2 revealing the presence of two distinct bands: a minor band with a low-molecular-weight form corresponding to a normal IGF2 and a major band with a high-molecular-weight form corresponding to an incompletely processed precursor of IGF2.

Hypoglycemic episodes lasted over subsequent days despite the continuous daily infusion of glucose and glucagon. Therefore, paraneoplastic cause of hypoglycemia was suspected on the basis of the MSFT history and the absence of a common etiology. The serum IGF1 level was low: 56 ng/ml (normal range: 87–195). Electrophoresis of plasma IGF2 revealed the presence of two distinct bands in the patient's serum: a minor band with a low-molecular-weight form corresponding to a normal IGF2 and a major band with a high-molecular-weight form corresponding to an incompletely processed precursor of IGF2 (Fig. 2). Thus, the diagnosis of a non-islet cell tumor-induced hypoglycemia (NICTH) was established on the basis of the hypoinsulinemic hypoglycemia, the MSFT history, the presence of an immature form of IGF2, and the exclusion of any other hypoglycemic causes.

## Treatment

Continuous daily i.v. administration of glucose solute, combined with i.m. injections of glucagon (1 mg daily) failed to achieve a perfect glycemic control even after four days. Therefore, therapy with corticosteroids commenced at a dose of 25 mg/day (0.5 mg/kg per day), after which her general status, consciousness disorders, and hypoglycemic episodes improved.

## Outcome and follow-up

Two days later, infusion of glucose and glucagon administration were discontinued and a normal glycemic control was achieved with prednisone alone. The patient left our department with prednisone 20 mg/day. A progressive decrease in the dose (5 mg/week) was expected during the outpatient consultation with a target to maintain the lowest effective dose (∼5 to 10 mg/day). Further, the patient was provided with a device and education for self-monitoring blood glucose.

## Discussion

Herein, we report a case of NICTH induced by an MSFT. NICTH occurs mainly in patients with solid tumors of mesenchymal and epithelial origins and, less frequently, in hematopoietic and neuroendocrine tumors (4). IGF2 is produced by the liver and interacts mostly with IGF1 and insulin receptors. The big IGF2 is an unprocessed high-molecular-weight form with a persistent insulin-like activity. Big IGF2 may also account for elevated glucose consumption in the tumor by autocrine and paracrine effects (4).

Hypoglycemia leads to the diagnosis of the tumor in 50% of cases (4). In other cases, hypoglycemia occurs after the tumor has been found (4). Usually, patients report previous hypoglycemic symptoms before the NICTH has been diagnosed (4). However, hypoglycemic symptoms can suddenly occur in other cases (5). Neuroglycopenic symptoms are more commonly observed than autonomic symptoms due to repeated hypoglycemic events and insidious progression observed with NICTH (4). In the present case, NICTH was established six years after the diagnosis of MSFT, and the patient reported at least one episode of hypoglycemic symptoms few days before the diagnosis of NICTH. Clinical presentation was particularly characterized by neuroglycopenic symptoms.

The diagnosis of NICTH is based on the findings of hypoinsulinemic hypoglycemia associated with the presence of big IGF2 (4). Data about the detection methods of big IGF2 are scarce. Size-exclusion acid chromatography has been considered as the gold standard method for the detection of big IGF2 in NICTH (4). This method provides good separation of big IGF2 from mature IGF2. Otherwise, the western immunoblot analysis has been shown to be a more rapid and equally sensitive method than size-exclusion chromatography (6). Electrophoresis of IGF2 is a semiquantitative, less expensive, and fast method.

The treatment of NICTH should target both a symptomatic management of hypoglycemic episodes and the tumor treatment. Hypoglycemia could be reversible after a successful tumor surgery (4). Chemotherapy or embolization can reduce the occurrence of hypoglycemia (4) (7). Glucocorticosteroids seem to be the most effective symptomatic treatment. They stimulate gluconeogenesis and inhibit the big IGF2 tumor production (8). This effect is dose dependent and reversible if doses are below a critical level (9). GH therapy relieves hypoglycemic symptoms although it fails to suppress tumor IGF2 production (7) (10).

## Patient's perspective

The patient passed away one year after she was presented to the Department of Endocrinology secondary to her MSFT.

## Author contribution statement

Dr K Mohammedi, the first and corresponding author, is the physician responsible for the patient. Dr K Mohammedi and Dr C Abi Khalil wrote the manuscript. All authors contributed to the discussion and reviewed the manuscript.
